# Practical Surgery

**Published:** 1846-10

**Authors:** 


### ART. VII.

1. *Practical Surgery.* By ROBERT LISTON. Fourth edition.—*London,* 1846. 8vo, pp. 582.

2. *Lectures on the Operations of Surgery, and on Diseases and Accidents requiring Operations.* By ROBERT LISTON, Esq., F.R.S., &c.; with numerous Additions, by THOMAS D. MÜTTER, M.D., Professor of Surgery in Jefferson College, Philadelphia, &c.—*Philadelphia,* 1846. 8vo, pp. 566.

I. IN the Fifth Volume of our Review, p. 457, we gave a somewhat lengthened analysis of the first edition of Mr. Liston's 'Practical Surgery,' and extracted what appeared to us the most novel and interesting of his observations. The two next editions were allowed to pass almost unnoticed, their rapid succession being a sufficiently decided expression of the spirit in which the work was received by the profession, and amply bearing out the very favorable opinion we originally passed upon it.

The appearance of a fourth edition reminds us that opportunity has been given for the further development of the work; and, accordingly, after a careful perusal of the new volume, we propose to lay before our readers the chief additions to facts, and modifications of opinion, which we find therein contained. Such changes are looked for with interest, as the result of several years' additional experience to a surgeon who occupies a prominent and distinguished place in the ranks of his profession, and possesses large opportunities of pursuing practically the subject on which he writes.

The object, plan, and general arrangement of the work remain unchanged; the amount of information contained within its pages has, however, been increased; facts have accumulated, and the illustrations of particular forms of disease and methods of treatment have been enriched by the addition of several interesting and pertinent cases, and many useful and admirably executed woodcuts.

Of the object the author has in view we need say little, and it would, indeed, be equally idle to insist here on the importance of the manipulative branch of surgery. Mr. Liston has the credit of having perceived and supplied a defect in the surgical literature of the country. Since the publication of Mr. Liston's work the deficiency it supplied has been pretty generally acknowledged, and more than one rival treatise has started into existence, proving, we believe, the increased attention drawn to the subject, and the greater anxiety of the junior members of the profession to fit themselves for the management of cases occurring in country practice, which, in years gone by, were at once and as matter of course transferred to the hands of the London surgeon.

Mr. Liston's volume may be described as a series of essays on the principal injuries and diseases which require manual interference on the part of the surgeon, together with directions as to the modes of proceeding which he has himself found most convenient and successful. As a guide to the advanced student, and as suggesting practical observations of the highest value to the practitioner, it is unsurpassed; though, as a complete text-book of practical surgery for the younger student, it is capable of improvement.

Mr. Liston tells us, in his Preface, what is and what is not to be expected from his work:

"The young surgeon ought, without doubt, to understand well the history of his profession, and this is only to be acquired by a perusal of the leading surgical works from the time of Ambrose Paré downwards to Wiseman and Heister. . . . . The reader of this work would not be much wiser, however, or more capable of undertaking the management of difficult cases, were the author to enter into a long detail of what this or the other of the moderns have recommended, or what he has found by experience in practice to be useless or hurtful. It has been the writer's aim to present a practical work, to which the young surgeon, when in difficulty, can refer, in hope of finding concisely stated and without conflicting opinions, that which he himself has, after a good deal of experience, seen and ascertained to be the wisest and safest course to pursue under various circumstances." (Preface, p. v.)

In the general spirit of these remarks we fully concur. We are no advocates of the practice, too prevalent in French Manuals, of cramming professedly practical works with detailed descriptions of the particular operation of this surgeon, the modification of that, or the proposal of another, for arriving at the same end. It may be well to put on record, and keep for reference, in dictionaries and encyclopædias, these numerous and often small modifications in practice, as proposed and carried into operation at various times and in different countries; and though practically many such propositions may have been long superseded by better modes of operation, they still form interesting records of the amount of intellect and attention turned to the subject. Thus collected, they will be consulted by all well-educated surgeons, and may serve sometimes as a means of humbling the pretensions of many a candidate, too eager for distinction, who would produce as a new discovery some exploded theory or practice. But to introduce such particulars into a work intended to guide a practitioner in the best management of particular cases, is to run the chance of producing confusion instead of giving confidence, and has the appearance, to say the least, of an ill-timed exhibition of learning. All this we are prepared to admit; yet we think Mr. Liston might have gone further into detail on some points: for although it would be a waste of time to detail everything "he has found in practice to be useless or hurtful," it might often be worth while to give concisely his chief reasons for adopting and recommending this or that particular mode of procedure in preference to any other. The young practitioner, who consults the works of a practical man more in the hope of finding a decidedly expressed opinion to guide and strengthen his judgment, than with the view of refreshing his memory on the history of improvements in practice, will not consider the weight of the opinion lessened by a short statement of the grounds on which it is based.

We venture to throw out these suggestions because we are anxious that a work so worthy of its author should have its utility extended; and we are convinced that a brief statement, perhaps in the form of notes, of reasons which decided the operator in preferring one method to another, would be invaluable both to the practitioner and to the student.

We now pass on to a more detailed examination of the chapters which have received additions or undergone some change.

Appended to the first chapter, which treats *of the division of parts by the knife, and the means of suppressing hemorrhage,* we find remarks on the employment of astringents and escharotics, in which the author points out the preparations he has found by experience best adapted to fulfil certain special indications.

> "Some will be found to serve one purpose well, others another. In fact, as far as I can apprehend, after some experience, every caustic has its own peculiar properties and uses, and no one of them can supply the place of others satisfactorily. If an escharotic is plainly wanted, the suitable one must at once be brought into play; they cannot with propriety or advantage be used indiscriminately." (p. 26.)

This is a point which may seem small and unimportant, but it is one which we have reason to believe does not receive the attention it deserves. On the one hand, it is too much the habit of would-be surgeons to trifle with caustics, and to apply them as a matter of routine practice in every case where they find a sore halt in the process of cicatrization; and, on the other hand, it is comparatively of rare occurrence for a practitioner to be called upon to use an escharotic to considerable extent, yet with an exact precision. The uncertainty of the action of caustics is, with many, almost proverbial. In one instance the surgeon is obliged to repeat the application again and again, in order to produce an effect he hoped to attain by the first use of caustics; whilst, in another case, he is perhaps annoyed to find, when the sloughs separate, that the soft parts have been more widely and deeply destroyed than he intended or expected. These are accidents which must frequently come under the observation of those who have an opportunity of witnessing much practice, and we are inclined to infer from them, not that the escharotics themselves are so uncertain in their action, but that there is very frequently a want of anything like a fixed principle to guide in their selection and application. Mr. Liston finishes his observations on this head by the following characteristic advice:

"In conclusion, if tissues are to be destroyed, let it be done at once, by the use of a proper and efficient caustic; and this is much better for all parties concerned than the practice of piddling from day to day with little or inefficient applications, as is often done in lupus and other diseases. The patient's patience is worn out, and the disease thus often deteriorated." (p. 28.)

In the chapter on *injuries of the bones* (parts of which, as on fractures of the spine for example, might with advantage be extended), we would draw attention to Mr. Liston's opinion on the importance of an early adjustment and elevated position in cases of fracture of the long bones. This he expresses in the following strong terms:

"The object of the surgeon in the treatment of all fractures must be to obviate pain and suffering, to put the parts in the most favorable condition for being repaired, and to preserve the limb of its normal shape and length. All these indications are fulfilled by the same means, viz. instant coaptation, and retention of the broken ends in the most perfect possible apposition. The earlier the means are adopted, the greater and more immediate will be the patient's relief from suffering, and the less the surgeon's anxiety and labour; excess of bloody and serous effusion will thus also be prevented, and the excited action kept within bounds. Prevention, it is admitted, is always better than cure; and if the above recommendation is attended to, all necessity for local abstraction of blood, and for the use of lotions to cool the part, will be obviated. If, on the contrary, the limb is laid loosely on a pillow, in an easy position, as it is by some thought and said to be, muscles relaxed, &c., and no efficient means are employed to prevent the spasmodic action of the muscles, the startings of the limb, the jerkings of the broken ends, and the displacement of the fragments, then assuredly, in spite of all local and general measures, there will arise frightful swelling, pain, tension, and heat; the intermuscular tissue will be gorged with blood, and the circulation of the limb roused to a dangerous and alarming degree. No application of soap plaster, in strips, gently around the limb, 'by causing secretion underneath it,' can reasonably be supposed likely to diminish either the irritability of the muscles or the urgency of the inflammation; nor will it. The splints, when resorted to, must be put upon a swollen and distorted member." (p. 62.)

Experience and observation have abundantly proved to us the propriety of this advice, and its soundness as a safe principle of treatment; though, in this as in every other case, particular exceptions may demand a deviation from the general rule. In country practice, for example, where possibly both patient and surgeon have to travel some distance before the injury can be attended to, where consequently a considerable interval is unavoidably allowed to elapse before the necessary materials for "putting up the fracture" can be collected, and where the habit and constitution of the patient render him more prone to the early occurrence of inflammatory action, the surgeon may sometimes find it advisable to resort, in the first instance, to fomentations, evaporating lotions, &c., the limb being elevated, however, and just so far confined as to prevent it from starting, or the ends of the bones from riding over each other. But in town practice, and generally in hospitals, where competent assistance is always at hand, and where the exact nature of the injury can probably at once be satisfactorily made out, we believe that the necessity for delay in the adjustment of the fracture and the application of splints is of extremely rare occurrence. It surprises one who has been in the habit of following this method to hear a surgeon of a metropolitan hospital recommend that a fracture should be left on a pillow for a few days, enveloped in strips of soap-plaster. We might ask what object is attained by the plaster that would not be equally well, or better, effected by the ordinary apparatus? A question of equally difficult solution is, what objection can be made to the application of splints and a moderately tight bandage, that does not equally apply to the employment of strips of plaster? The young surgeon who has been taught to expect the supervention of inflammatory symptoms as the natural consequence of breaking the bone, is rather confirmed in his erroneous impression by finding that, in spite of what he is led to believe the best precautions against it, inflammation sets in. It perhaps never occurs to him to suspect that the very precautions he observes may tend to produce that result. Let him, however, treat an equal number of cases on the principle of immediate adjustment and elevated position, and we feel convinced he will be surprised how seldom the evil he apprehends will actually manifest itself.

We must refer our readers to the work itself for much interesting matter in the chapter on *injuries and diseases of joints.* The indications of treatment are laid down clearly, and the subject is handled concisely and with strong common sense. One division of the chapter is headed "Resection of Bones," but the only operations described are those on the elbow and shoulder-joints. The methods of proceeding for the resection of the upper and lower jaws and clavicle are detailed when treating of tumours and morbid growths; and excision of the articular surfaces composing the knee and hip-joints, is not considered by Mr. Liston sufficiently encouraging in its results to demand any detailed notice from him. The value of the operation as applied to the elbow and shoulder-joints, is now generally admitted, though there is still a want of statistical information (more particularly as regards the arresting diseased action and preserving the after utility of the limb), a deficiency which can be supplied only by following up the history of persons so treated for several years after the operation. It would, however, appear to be established by facts, that the result of excision of the joints is most favorable in children; and this we think might have been expected. Nature at this early period of life has more power to repair extensive injuries, and to make up for deficiencies produced either by the ravages of disease, or by the means taken by the surgeon to arrest its progress. The material by which the uniting of the several ends of the bones is effected, after excision of the joint of a child, is more elastic and pliable, and forms a better substitute for a capsular ligament than the tough and irregular deposit that forms the medium of connexion in the limbs of older persons. The development of the parts, the growth of particular muscles, and the habits of the patient in using and managing his limb, are all probably more easily and thoroughly modified in the instance of a young person. Even in an adult, however, under favorable circumstances, a very serviceable limb may be the result of the operation; and with a careful selection of cases, there is every reason to expect that the operation will continue (as it has already done for the last few years) to rise in the estimation of practical surgeons. Happily, it is not so likely as some more brilliant operations to be too generally or indiscriminately employed. In the case of the elbow, for example, the method of excision is so much more tedious and painful, so much more difficult of execution, and so much more severe in appearance than amputation of the arm, that the greater number of surgeons naturally lean towards the latter alternative, without perhaps sufficiently considering that the patient may feel amply repaid for extra suffering in retaining his forearm and hand.

Any undue preference of this kind will be gradually corrected as our information on the subject accumulates, and in the meanwhile we believe that the operation of excision has a better chance of being appreciated than if, instead of being received with hesitation, it had at once been over-praised and indiscriminately adopted. It might be said that excision of the joints has retrograded in consequence of being originally thus overvalued, because this method of treatment remained for many years comparatively unnoticed, until again urged upon the attention of the profession by Mr. Syme. But we conceive that other causes have been in operation to produce a fairer estimate of excision; for, as now modified and limited in its application, that mode of treatment is already on a very different footing from that on which it was placed by Park and Moreau. Speaking of the circumstances under which the operation has the best chance of success, Mr. Liston remarks:

"Such operations may be resorted to with propriety when the patient's general health has not suffered much, or when, from keeping the joint in a state of quietude, the discharge has abated, and the system recovered from the effects of the irritation and constant drain. When, however, the soft parts are much diseased, when, as is often the case, the disease is not limited to the articulating surfaces, or when the patient is reduced to a very low state by hectic, it may not be very prudent to try the experiment; for it has happened that, after all, the proceeding has not been followed by removal of the whole disease, or subsidence of the constitutional disturbance; and in the end amputation has been deemed advisable. And this latter operation, which alone and in the first instance, might have been borne up against, has at that stage been followed by a fatal result." (p. 152.)

Excision, as employed in the case of the elbow, is seen in its most favorable application. It should be borne in mind that this joint is, for many reasons, particularly well circumstanced to ensure success. As regards the operation, the elbow is so situated and constructed as to render comparatively easy the full exposure and safe removal of the diseased surfaces of bone; and in reference to the final result a very useful limb is preserved, even in cases where but little motion remains in the new joint.

In performing this operation Mr. Liston always adopts the same lines of incision. The first is made in the direction of the length of the limb, and close to the radial side of the course of the ulnar nerve; the second extends from the centre of this incision, across the joint, to the outer condyle, and the two flaps marked out are then dissected from the bones and turned upwards and downwards. The advice given by some writers, to expose the joint by slitting up sinuses where they exist, appears to us more plausible than sound. Such a proceeding saves the patient very little, if any, pain; it may seriously hamper and embarrass the surgeon in the subsequent steps of the operation; and, lastly, it is not absolutely required, because all these sinuses readily and rapidly close when the cause of irritation is removed, and when a more direct and free exit for the discharge of pus is established. The soft parts being reflected from the bones, Mr. Liston directs that the forearm should be flexed on the arm, and the ligaments at once freely divided. In this position he finds no difficulty in dislocating the bones, and examining the surfaces which enter into the formation of the joint, without first sawing off the end of the olecranon, as practised and recommended by Mr. Syme and others.

Cases are recorded of excision of the elbow, even in adults, in which the use of the forearm was retained in an extraordinary degree; and so far they would tend to encourage the early employment of passive movement of the limb after the operation. It is possible, however, in attempting too much, to lose all the benefit of the operation; and, considering the insufficient data we at present possess, it must be regarded as a delicate question how far the surgeon should aim at reestablishing the motions of flexion and extension of the forearm. On this point Mr. Liston appears to us to convey a wholesome caution, by not speaking in quite such sanguine terms as some writers have done:

"Unless the operation on the elbow be performed when the patient is very young, and the portions of bone removed be very small, the motions of that joint must of necessity be ever after very weak and unsteady. In the operations which I have performed on the adult, amounting in all to eight, for thorough removal of the ends of all the three bones, I have aimed at bringing about a kind of ligamentous anchylosis, by steadying the part for a long time by means of leathern splints. In fact, the patient, in order to use the forearm and hand to any advantage, will require, for a time at least, to have some permanent contrivance to fix the forearm at a right angle with the upper arm." (p. 152.)

"In more favorable cases the patient will acquire very considerable power of motion in the false joint. He will be able to straighten and flex the arm almost as completely as before the operation. The union in these cases is not usually effected by anchylosis, but a dense fibrous tissue passes between the ends of the bones at all parts, and the whole is surrounded by strong ligamentous substance, to which the muscles are inseparably united. The fibrous tissue between the bones is longer or shorter as more or less motion is permitted." (p. 154.)

In Chapter VI, *On injuries and diseases of blood-vessels,* some interesting and instructive cases will be found of hemorrhage from ulcers and abscesses, produced by an extension of ulceration, from without inwards, through the coats of the larger vessels. Both arteries and veins are subject to this accident; but the latter class of vessels are more frequently so affected in the case of ulcers. Several instances, however, have been collected by Mr. Liston of perforation of the coats of arterial trunks and branches, to the extent of effecting a free communication with the sac of an acute or chronic abscess; and these have been noticed by us on a former occasion. (British and Foreign Medical Review, Vol. XV, p. 155.) The following case, which occurred in the practice of Mr. Cheyne, of Leith, is added:

"H. R., æt. 6, Nov. 1828, was treated for chronic peritonitis. Two years before this attack he had suffered from fever, with pulmonary symptoms, for five weeks. After recovering from this disease, he enjoyed tolerable health till within four weeks of his death. He was then seized with remittent fever, but no region or organ appeared particularly affected till the end of the first week, when the belly rather suddenly became tympanitic, and the child complained much of pain on pressure. The remissions became more distinct; and during the fourth week a dry and rather severe cough troubled him. At the end of this week, one day about noon, he vomited a large quantity of blood; rallied a little; but in an hour after, upon vomiting more blood, he rapidly sank. On dissection, the peritoneal surface bore all the marks of intense inflammatory action having existed; the membrane was much congested and thickened. In the stomach about a pound of clotted blood was found. At the upper part of the descending aorta there was a small ragged perforation, leading to a cavity that might contain a walnut; this cavity, filled with coagulated blood, communicated with the œsophagus by an orifice capable of admitting the little finger. It was situated in the midst of the bronchial glands, which were more or less changed into a texture of a cheesy appearance. The lungs adhered pretty generally to the thorax by bands of old formation. In their substance there was a yellow tuberculous mass, of the size of a marble, and a few smaller ones in its vicinity." (p. 192.)

After describing the operations for applying a ligature to the external iliac and femoral arteries, Mr. Liston mentions the treatment of aneurism by pressure over the arterial trunk at some convenient point between the tumour and the heart, in the following terms:

"Within the last few years the treatment of aneurism of the middle of the thigh and ham by pressure, in a modified form, has been revived with very encouraging results. In all, some ten or a dozen cases, have up to this time (1845) been so managed. The practice was first employed by Mr. Hutton, and followed by some of the Dublin surgeons. A few cases have also been cured in this country; two of them under my care in the University College Hospital. The object in applying the pressure is not to stay entirely the current of blood, but merely to weaken it, so as to permit coagulation of the contents of the sac to proceed gradually until this process is completed. For this purpose the femoral artery has applied over it a pad secured by a sort of C-shaped piece of steel, jointed in the middle, and with a larger pad by which counter-pressure behind is effected..... The skin over the vessel should be protected with plaster on the back of the thigh, by a well-fitted leather splint. The patient may have almost the entire control over the instrument, screwing it up or relaxing the pressure according to his feelings. It is by no means a bad plan to apply two such tourniquets, which can be tightened or relaxed alternately. By adopting this method a cure may pretty confidently be expected, and the patient is certainly saved from considerable risk, as from secondary hemorrhage and inflammation of the vein." (p. 222.)

The advantages of the treatment by pressure appear to us to be by no means limited to the avoidance of the dangers which are incurred by the application of a ligature in the usual manner; such as secondary hemorrhage from the separation of the ligature, and phlebitis. In the first place, the plan by pressure is one likely to be much more readily submitted to by patients than what is known to be a more severe and hazardous, and is believed (although erroneously,) to be a more painful proceeding: and thus not only may many cases be brought under treatment that would otherwise be allowed to advance gradually to a fatal termination, but many more may be attacked at an earlier period than is now permitted under the contemplation of a different alternative.

The general principles on which the treatment by pressure is now based are so simple, and the precautions necessary to be observed so few and easily understood, that we may state facility of application as another recommendation—not, indeed, to the accomplished operator, but to the more numerous class of surgeons who, with fitting cases for pressure under their charge, are unwilling to grapple with the difficulties of an operation which requires in the operator a practised hand and confidence in his knowledge of the anatomy of the parts concerned.

Again, the mode of treatment by pressure has this advantage: it is capable of being interrupted for a time, or entirely stopped, if necessary. On the other hand, when a ligature is applied on the main trunk of an artery the step is irretrievable; and if the arrest of the circulation through the limb should prove to be too complete, and the usual signs and symptoms of mortification should in a few hours begin to appear, there is little to be done but to await the more unequivocal indications of failure, with the alternative of mutilation, or almost certain death, should the symptoms increase. Lastly, there is every reason to regard pressure as so safe a proceeding, that it may be applied in instances where the attendant circumstances forbid a more hazardous course of treatment.

It is perhaps rather premature to affirm that, even should the treatment by pressure fail, the patient is left in no worse position than before the attempt. The number of cases at present on record is hardly sufficient to justify so unqualified an assertion. If the case be of such a description that an operation by ligature is considered unjustifiable, the failure of the plan by pressure to produce a cure certainly implies no aggravation of the evil; whilst so far as even a temporary arrest of the progress of the disease has been effected, the patient must be considered to have gained by the treatment. But if, on the contrary, the case be of a nature which offers, at first, a fair prospect of success to the operation by ligature, it is possible that long-continued compression may produce such an alteration in the condition of the main artery, and of the parts immediately surrounding it, between the seat of pressure and the tumour, as to throw difficulties in the way of the subsequent application of a ligature, which would not have existed had not pressure been resorted to. And this leads us to remark that in cases of the kind last referred to, it may be a principle worth holding in view to apply the pad of the instrument at such a point as to leave sufficient room above for the proper application of a ligature (should such a procedure be found, after all, advisable) to a portion of uncompressed artery.

One of the most interesting and valuable parts of the volume is that which is devoted to a consideration of *morbid growths and enlargements.* As the principal object is the description of the variously modified operations that are required for the removal of these growths from different situations, Mr. Liston classes tumours according to the localities in which they arise; and only touches incidentally upon their differences in structure and general character. Speaking of some of these diagnostic signs between simple and malignant tumours—between those he would remove and those he would leave untouched, Mr. Liston takes occasion to reprobate, in strong terms, the frequent and indiscriminate use of grooved needles and exploring trocars:

"The surgeon, by studying well the nature and progress of growths in this situation (the upper jaw), and, in fact, in all parts of the body—by losing no opportunity of making examinations, and thus gaining the requisite tact, will be able to predict their internal structure with tolerable certainty. He will not be under the necessity of resorting to what, in many cases, proves a very mischievous and hurtful influence, the thrusting into their substance of a long, flat, or round and grooved needle. I was consulted very lately by a gentleman labouring under a tumour occupying the floor of the antrum, who had shortly before suffered very severely in consequence of this idle curiosity; the action of the diseased part had been roused, and for some time after considerable increase of bulk had taken place. A day or two after this there was presented at the hospital a case of tumour over the distal end of the ulna, of firm consistence and very painful nature, involving and compressing the dorsal branch of the ulnar nerve. There was cicatrix resulting from a punctured incision upon the skin covering it. The nature of the case was represented to the young woman, and extirpation recommended. She returned to have this done in two days, with a fresh mark of puncture upon the tumour. The operation was performed, and inquiry then made as to how this second mark had been made. She confessed that she had, in the interval, gone to another hospital for advice, had there the swelling explored, and suffered in consequence, as she declared, much more pain than that caused by the extirpation." (p. 314.)

Mr. Liston, however, admits the value of the information derived from this mode of examination, and countenances its employment in obscure cases:

"I had recourse to the practice lately in a very obscure case. A middle-aged woman was admitted into the North London Hospital, labouring under a large tumour situated in the lower part of the abdomen, and filling the pelvis. The bladder was displaced, and occasional retention of urine took place; difficulty was always experienced in introducing a catheter, and it was necessary to depress the handle so as to convey the point of the instrument close to the symphysis. Indistinct fluctuation, it was supposed, could be detected by pressing alternately on the tumour above the pubes, one finger being placed on the projection in the vagina, the other on the external swelling; the os tincæ could neither be felt nor seen by the use of the speculum. A large grooved needle was introduced into the vaginal tumour, and a small quantity of pus was perceived. This was immediately followed by a large flat trocar, and nearly three pints of well-digested pus evacuated. The patient ultimately died; the cyst was found connected with one of the ovaries, and contained, besides matter, a large ball of hair—no uncommon occurrence." (p. 315.)

We have not room for more than a passing reference to Mr. Liston's directions as to the various operations for the removal of tumours. The manner in which the whole subject is handled is well calculated to impress the reader with the conviction that the author is speaking from large personal experience and frequent trial of the particular modes of practice he recommends.

The first object in the performance of these irregular, and often extremely perplexing and difficult, operations is to make sufficiently free and extensive incisions through the skin, to ensure the complete exposure and most ready dissection of the chief attachments of the morbid growth; and this "good beginning" is all-important, not only because it really shortens the suffering of the patient, by giving every possible facility to the after steps of the operation, but because it enables the surgeon to satisfy himself more thoroughly as to the entire removal of the diseased tissues. The next object, and one often of no slight importance, is so to arrange these incisions as to leave as little disfigurement as possible, either from loss of integument or from unseemly and conspicuous scars.

These considerations seem constantly present in Mr. Liston's mind; and much ingenuity is displayed in his plans to carry out both objects. A reference to his directions for the removal of parts of the upper and lower jaws, on account of tumours affecting the bones, and ligature of erectile tumours not involving the skin, will sufficiently illustrate these remarks.

Nothing is said yet of the plan for the *subcutaneous* application of ligatures, as lately proposed in Paris by MM. Ballard and Rigal, for the destruction of tumours in the thyroid gland, and applicable, it may be supposed, to cases of nævus. We can readily conceive, however, that the cases which would admit of this somewhat more complex proceeding are by no means numerous or common.

The chapter on *amputations* has received further illustration by several new and well-planned woodcuts, but is otherwise little altered. Mr. Liston is well known to be a strong advocate for the method by flaps, and for leaving the dressing of the stump until all oozing of blood from the cut surfaces has ceased. In amputation of the leg he seems to prefer dividing the tibia and fibula to separating the limb at the ankle-joint; the latter operation, though lately so much recommended, is not described or noticed by him. Amputation at the elbow-joint is advised in cases of injury involving a great part of the forearm.

Several good cases will be found in the next chapter, illustrating the *lodgement of foreign bodies in the windpipe*, &c.; and a successful case of extraction of a button from the bronchus is also recorded. The patient, a boy eight years old, allowed the button to slip into his trachea whilst at play, and was instantly seized with extreme difficulty of breathing, threatening suffocation. On the tenth day after the accident, the irritation having in a great measure subsided, and simpler means having failed to dislodge the foreign body, the windpipe was opened by Mr. Dickin, of Middleton, the opening extending from the crico-thyroid membrane downwards for about an inch. A pair of long and slightly bent forceps being introduced, the foreign body was struck, and, on a second attempt, seized and extracted. Another case is related, in which a bullet was dislodged from the bronchus by the sudden inversion of the patient, who was strapped to a common chair and slung to the rafters of the ceiling. He was turned over three times and repeatedly shaken, and the third time the bullet rolled from his mouth.

*Lithotrity.* Though known to be a very skilful and successful lithotomist, Mr. Liston appears to have recognised and practised the operation of lithotrity at an early period; and, as far as we can judge from the terms in which he speaks of this mode of treatment, his confidence in its efficacy, within certain limits and under certain restrictions, is increased by additional experience, and by the improvements that have been effected in the construction of the crushing instruments. After shortly comparing the advantages of each plan under different circumstances, he adds, "of late years I have scarcely been obliged to have recourse to lithotomy at all in private practice." It is hardly necessary to remark that such an admission is of peculiar value, and comes with additional weight from a man who, with large experience and a high reputation as a lithotomist, may naturally be supposed to have a certain, even unconscious, leaning towards an operation in which he feels his strength, and of which the difficulties are universally acknowledged. Mr. Liston's estimate of the value of lithotomy and lithotrity is, we think, fairly and candidly stated. The accidents that may follow the operation of lithotrity, far from being overstated, might with advantage be dwelt upon more fully. These accidents—including inflammatory congestion of the parts around the neck of the bladder and membraneous portion of the urethra, hemorrhage, cystitis, impaction of fragments in the neck of the bladder, and distressing spasmodic action of the parts in the neighbourhood, retention of urine from this latter cause, or from the presence of coagula in the bladder, or from partial paralysis of that viscus, arrest of pieces of calculus in the urethra, swelling of the testicle, and even suppression of urine—should all be fully considered, not only for the purpose of detailing the appropriate means of treatment, but with the view of fairly setting before the surgeon who undertakes lithotrity the accidents which he may meet with, and must be prepared to avoid or overcome. The two methods of operation, as witnessed in one or two ordinary and perhaps successful cases, leave very different impressions of the dangers to which the patient is exposed—impressions which, we may add, are quite disproportioned to the real risk incurred. French lithotritists have, with great power, drawn a vivid picture of the horrors of lithotomy, in order, apparently, to induce to the belief that lithotrity, because it does not present to the eye all those startling accompaniments, is without its dangers. But this is a short-sighted policy; even assuming that these supporters of lithotrity are thoroughly convinced that the measures they recommend are much more frequently applicable than is generally supposed. This dingenuousness, as is the case with every, even the slightest, exaggeration or suppression of facts, produces a twofold mischief. The immediate effect of inculcating such prejudices on an inexperienced mind may be to lower the estimation of lithotomy; but the ultimate effect (and one is sure to follow the other) is an equally unjust depreciation of lithotrity, when it is found unexpectedly that a surgeon may, in attempting to crush a stone, occasion a more serious mischief than perhaps at once appears. In order that strict justice should be done to lithotrity, no less than that the surgeon should be able to apply it judiciously and effectually, the unfavorable as well as the favorable side of the picture should be exposed, and the operator put on his guard as to the difficulties he may encounter.

The chapter on *hernia* we only refer to in order to point out a decided advance in opinion on the subject of dividing the stricture in cases of strangulated hernia, without opening the sac—a change of opinion which is based on the most satisfactory of all foundations, namely, actual experience after a fair and judicious trial. All who are aware of the frequent occurrence of peritonitis after the ordinary operation for strangulated hernia, and reflect on the causes chiefly concerned in such cases in the production of so unmanageable and fatal a disease, must admit as important and reasonable the proposal made by Petit, considerably more than a century ago, of avoiding, if possible, the exposure of the peritoneal surface. As limited in its application by Sir Astley Cooper, namely, as applied to old and large hernia, the principle is now recognised by most surgical authorities; but the further extension of the principle, as argued for by Petit and Monro, and more recently by Mr. Key and others, has met with strenuous opposition from some, and neglect from many more, who were content, perhaps on theoretical rather than on practical grounds, to throw doubts upon the soundness of the suggestion. First, the possibility of effecting the object, then the expediency of making the attempt, was called in question; and the result has been to delay the period at which, after a sufficiently extensive trial, an impartial judgment might be passed upon the practice. However, there are few improvements in medicine or surgery the expediency of which has been, from the first proposal, so manifest as to have escaped altogether a similar opposition. Indeed there may be said to exist a kind of professional *vis inertiæ* which can be overcome only by degrees, because the objections it raises must be met, not by argument, but by facts; and these require time to be collected in sufficient number.

Six years ago Mr. Liston expressed himself doubtfully, and, on the whole, unfavorably, of the plan of dividing the stricture without opening the sac. "It is," he remarked, "rarely possible to effect the object, although there is no harm done by trying it in some cases; but in others, constituting the vast majority, the attempt must prove very unsafe." Now, however, after a longer trial, he recommends the method more decidedly and unhesitatingly. After describing the preliminary incisions for exposing a strangulated femoral hernia, for example, he proceeds as follows:

"If the surgeon aim at trying to give relief without opening the sac, as he always ought to do in cases of recent strangulation, he will now pass his narrow blunt-pointed bistoury betwixt it and the fascia, upon a director insinuated under the sharp and tight margin of the crural aperture (the director may often be dispensed with); then turning the edge forwards slightly towards the mesial line, and raising its handle, he will divide the resisting fibres. He may now try to reduce the contents of the sac by pressure, taking especial care not to push back sac and all—rather a serious accident. . . . . . I have fortunately succeeded in effecting this object in a considerable number of instances within these few years; and it is a proceeding which I most strenuously advise the adoption of when nothing contraindicates it. The attempt can do no harm, it causes little or no delay; and if it is not successful, the sac, after all, is opened, and the operation completed. If it does prove successful, the surgeon's mind is relieved of an uncommon load of anxiety." (p. 557.)

In conclusion, it is scarcely necessary to repeat our earnest recommendation of Mr. Liston's work. Having on a former occasion expressed ourselves strongly on the subject, we can only add, that the present edition is, as it should be, even more worthy of our praise than its predecessors. The already practised surgeon will know how to value it; but the student and young practitioner stands more in need of a little warning to prevent him from forming a false estimate, not indeed of such works, but of his power to profit by them. The simple perusal of works of this high order, unless the student of surgery is careful, at the same time, to test his dexterity and correct his ideas by frequent and repeated trial on the dead body, will assuredly only prepare disappointment for himself and others. We are led to make this remark from having observed a tone which, more or less, pervades the whole of Mr. Liston's work, namely, that of rather under-estimating the difficulties of the measures he recommends. In one sense it would be unfair and unjust to assert that Mr. Liston's book is calculated to encourage an undue love of operating. A reference to his observations on excision of ovarian cysts, operations on cleft palate, division of muscles in cases of deformity of the spine, removal of malignant tumours, and many plastic operations, will satisfy the candid reader that he speaks much less sanguinely of the results of surgical interference in what may be called doubtful cases, than many writers on operative surgery. There is, however, a difference between sanctioning the attempt at interference in cases that ought not to be meddled with, and affording to ill-qualified persons a certain encouragement to undertake a method of treatment which, though in itself perfectly justifiable, they are not prepared to carry out. The terms "easy" and "difficult," as applied to different manipulations, may, when measured by the writer's own standard, be perfectly justifiable, and yet may mislead the inexperienced reader, and tend to engender in him a wish to operate, founded on a false confidence in his own judgment and skill. We believe that there are many, and we fear the tone we deprecate is likely to increase that number, who, when they know the kind of operation called for in a particular case, and can repeat methodically the different steps of the proceeding, feel themselves competent to undertake it. To these we can only say, in the words of our universal authority, "If to do were as easy as to know what were good to do, chapels had been churches, and poor men's cottages princes' palaces."

II. The second of the two works, the titles of which stand at the head of the present article, is a republication, "with numerous additions," by Professor Mütter, of Philadelphia, of some nineteen Lectures delivered by Mr. Liston, at University College, London, and reported in the 'Lancet,' during the years 1844 and 1845.

We have already said enough to convey an idea of the respect with which we regard Mr. Liston as an authority on the subject of operative surgery.

These Lectures, collected from the above source, embrace indeed many of the topics just noticed in his treatise on 'Practical Surgery;' they are treated here with much spirit; are illustrated by cases in the lecturer's hospital practice, which must have been under the observation of many of his hearers; and are occasionally enlivened with a somewhat freer discussion than he allows himself in his more formal work, of the lines of practice adopted and recommended by other surgeons. The style is characteristic, forcible, and well adapted to excite interest and arrest attention. Several points are only slightly touched on, or are passed over altogether; some of these are taken up by Professor Mütter, who thus adds [within brackets] about two hundred and fifty pages to the general body of the work.

Except typographically, we cannot say that these two elements are dexterously blended. Some of the additions are themselves excellent and highly practical; but, taken together, there is a want of harmony with the Lectures themselves, not only in style (which might be expected), but in tone and spirit, in arrangement, and in the mode of carrying out the plan. Though, of course, the result of much reading as well as of actual practice, the lectures of Mr. Liston are stamped with a certain individuality; the lecturer so contriving to look at and weigh even the discoveries of others, through the medium and by the scale of his own experience, that the whole of the information imparted comes with much of the freshness of originality—a quality which does not generally characterize the additions of Dr. Mütter. The frequent recurrence of phrases, such as the following: —"The great majority are in favour of as a general rule;"—"Most surgeons prefer;"—"The weight of authority is in favour of;"—"It is considered best;"—or, such and such circumstances "render this operation a favorite with most surgeons;"—give a character of feebleness to the writing, which no one who is acquainted with Dr. Mütter's high reputation as an operating surgeon, would think in any way applicable to the writer. Nor is this effect lessened by the detail of measures which are afterwards unceremoniously dismissed with the remark, that the proposal is merely mentioned "as one of the novelties of the day, and wholly unworthy of our confidence;" or again, that "no surgeon of any note would condescend to employ so puerile a means;" or, "it will soon be remembered merely as one of the idle whims of some inventive genius."

The Lectures are rapid sketches of the principal points of interest in a wide field; many of the additions are representations, begun on a more elaborate plan, but hurriedly finished, and less striking in the general result. Mr. Liston, it may be observed, presupposes a certain amount of knowledge of the general principles of surgery, as already given at a former part of the course by his colleague, Professor Cooper; and to this previous instruction he takes occasion to refer from time to time, when he passes lightly over abstract points, and adheres more closely to subjects of a strictly practical nature. Dr. Mütter proceeds on a different plan, and divides his subjects elaborately, as if giving the outline merely of a complete treatise. We can conceive two methods of oral instruction on such a science as surgery, each useful in its way, and yet each on such a perfectly distinct plan as to make it difficult (and only possible in an extended series of lectures) to unite the benefits of both. According to one plan, the lectures might consist of clear and graphic sketches of disease and its appropriate treatment, such as it exists in what may be called its typical forms, and always so connected with the relation of actual cases as to arrest and fix attention by rousing the hearer's sympathy. According to another plan, lectures might be made use of, less as a means of imparting particulars and minutiæ, than as an opportunity of directing the mind of the student in the exercise of thought; of pointing out to him the best and most credible sources whence to derive facts on the several subjects which present themselves; of giving him the valuable habit of arranging his knowledge in the best and closest possible manner, and of helping him to analyse, and to form a just estimate of the comparative value of information derived from different sources. These two objects might, indeed, be borne in mind at the same time, and carried out together by one lecturer, step by step; though we doubt the possibility of its being done in an ordinary course, in which a vast quantity and variety of matter is crowded. It would be a work of still greater labour to blend well together the results of two different minds directed to such different ends. We believe that Professor Mütter, therefore, was in error in wishing partially to combine elements of so different a nature and so difficult of combination; and in attempting to make these lectures (what they were never, to all appearance, intended to be) a complete and independent work.

The most important and interesting of the additions made by Dr. Mütter are those on the subject of plastic and other operations for the remedy of deformities and deficiencies, both congenital and acquired, of the soft parts. As the arrangement followed by Mr. Liston is based merely on the locality of the surgical diseases of which he treats, these observations and details of special operations are necessarily scattered through the work. This branch of operative surgery has been cultivated most perseveringly and with great success, by several surgeons in America, amongst whom Professor Mütter occupies a prominent position. His contributions to science, on this head more especially, are as important to the profession as we learn his practice has been creditable to himself and beneficial to those who have fallen under his hands.

The principle most clearly established by the repeated and varied trials of plastic operations appears to be that of making as broad and even a pedicle as possible to the transposed flap of skin, in order to leave an efficient vascular and nervous communication between the disturbed and the comparatively non-disturbed parts. The method of twisting the flap upon itself (as in the Indian operation for the formation of a new nose) has, we believe, been adhered to longer and carried out further in this country than the success attendant on it has warranted. A much more successful plan (as Dr. Mütter shows from his own experience and from that of other surgeons) consists in cutting the flap in such a situation, that the direction it is made to assume is inclined at only a small angle from that of the surface from which it is taken. The pedicle need not then be twisted, and may be made broader than when this is requisite; or, still better, where it is practicable, separating the deep attachment of the skin, for some distance on either side of the cicatrix or part to be filled up, sliding the then moveable integument over the subjacent parts until the defective surface is completely covered; and, lastly, securing it in its new position by the usual means. As an illustration of this principle, we may follow Professor Mütter in some of his descriptions of plastic operations.

In the fifth Lecture, when treating of the mode of partially restoring the alæ of the nose, we find the following account of an operation performed by the Professor in the case of a gentleman who had lost "the whole of the right ala, as well as the adjacent soft parts, as high up as the os nasi of the same side," leaving a transversely oval opening, which measured three quarters of an inch across and half an inch in its vertical diameter.

"Seating myself in front I commenced the operation by making, with a small convex-edged bistoury, an incision extending from a few lines above the *superior* border of the orifice, to a short distance *below* its inferior, and directed *downwards* and *outwards*. It did not penetrate to the bone, but was sufficiently profound to allow a flap about *three* lines in thickness to be readily detached. This incision was completely on the *outside* of the cicatrix, a portion of which was subsequently removed, in order to prevent its hardened edges from irritating the raw surface of the flap, which was to be placed immediately upon it. One or two arteries were cut across, but the hemorrhage from them was arrested by pressure, until the *second* incision was made. This commenced at the *terminal* extremity of the first, *and extended horizontally outwards about an inch.* A *triangular* flap was thus marked out, and immediately detached from the subjacent bone by dissecting with the edge of the knife, held nearly parallel to the surface of the cheek. In the execution of this part of the operation two or three arteries of some size were necessarily cut across, and required the application of the ligature. The *third* incision, which extended from the *initial* extremity of the *first* to the point of the nose, was made with a pair of strong scissors, those being preferred to the scalpel, in consequence of this margin of the orifice being, to a certain extent, loose and unsupported. The triangular piece of cicatrix included between the superior extremities of the first and third incisions, was then removed with the scalpel and forceps, and the sharp margin of the inferior portion of the opening also pared off, for reasons already stated. The hemorrhage having been arrested, and the parts properly sponged, the next step of the operation was undertaken. This consisted in the approximation of the first and third incisions, and the application of such measures as were calculated to retain the flap in its proper position. From the free dissection and the yielding character of the subcutaneous cellular tissue of the cheek, no difficulty was experienced in placing the edges in contact; and in order to ensure their perfect and close approximation, *four* stitches of the *interrupted* suture, made with saddler's silk, waxed and doubled, were passed. In addition, two or three small adhesive strips were applied to the spaces between the sutures. Finally, in order to prevent adhesion between the *flap* and the raw surface beneath it, and to give a better shape to the former, a small roll of soft lint, well oiled, was introduced into the *new nostril.* (p. 173.)

The sutures were not removed until the end of the fourth day, when union by the first intention was found to have taken place throughout. About the third week the lower margin of the flap was cut with a pair of scissors to give "the proper curve" to the ala. In consequence also of the septum of the nose being a little drawn to one side, apparently by the contraction of the flap, "the line of union between the base of the flap and the cheek was divided, cutting from within with a small scalpel, held parallel to the surface of the cheek, to the extent of three or four lines." The plug of lint also was increased in size, and a strip of plaster carried across from the tip of the nose over the sound cheek, so as to draw the whole organ in the opposite direction to that towards which it was inclined.

"At the end of the sixth week from the day of the first operation. it was determined to execute the 'third step' in the treatment. This consisted in the division of the skin and cellular tissue at the base of the flap, in a *semicircular* direction, the convexity of the curve looking outwards. The object of this incision was to give the peculiar rounded margin of an original ala, to diminish the fulness of the cheek where the natural depression should exist, which depression had of necessity been destroyed by the tension of the flap, and to permit a return of the perpendicular position of the deviated septum nasi. The incision was made with a small scalpel, and extended to the bone. In order to prevent the union of its margins, a small roll of oiled lint was introduced into the cut, and a strip of adhesive plaster applied to the tip of the nose, and fastened on the cheek of the sound side. . . . . . On the third day the dressings were removed, and it was found that the margins of the incisions were nearly cicatrized and beautifully rounded off. . . . . . This latter change was a very favorable circumstance, as it produced a depression in the exact spot at which it was required, in order to give a proper expression to the face. Had it not taken place there would have remained a *sort of inclined plane* from the bridge of the nose to the outer portion of the cheek. At the expiration of the ninth week my patient returned home with scarcely a vestige of his deformity remaining." (p. 176.)

Truly, American surgeons have very far outstripped their English brethren when they can take into account the "proper expression" of the face. We can, from this, better understand the contempt with which another of their surgical writers, remarking on the subject, designates "the fruits of the dexterous manipulations of most nose-makers" as being, "most frequently, little else than pug-shaped, flabby, and moveable knobs, that look more like small shrivelled potatoes than *bonâ fide* human noses."

Under the head of cleft-palate, concerning the operations for the relief of which Mr. Liston speaks very discouragingly, Dr. Mütter introduces some admirable, short, and practical observations on the chief sources of difficulty and danger, both at the time of and after the operation; together with a brief detail of the means available for obviating the effects of such untoward and often perplexing circumstances. Dr. Mütter lays great stress on the advantage of simplicity in the form and construction of instruments to be used in these operations, and on the expediency of frequently handling the parts, for some days before operating, so as to "familiarise" the fauces to the presence of foreign bodies.

In the eighth Lecture is introduced an interesting account of a case of cancer of the lower lip, in which Professor Mütter removed the diseased parts, and at the same time restored the lip by a plastic operation. The latter step, which consisted in detaching and drawing up from the chin two symmetrical side-flaps, which were then united along the middle line, was ingeniously planned, and appears to have succeeded admirably. The patient remained well when seen after an interval of two years. We doubt, however, whether surgeons in this country would not have been satisfied, in the first instance, with getting rid of the disease, and deferring any further proceeding until the tissues near the excised part should have cicatrised, and remained for some considerable period free from any appearance of return of the disease.

The treatment of deformities resulting from burns is also much illustrated in this volume by Dr. Mütter. The author, as is well known, has published, in a separate form, several valuable essays on this subject; but as the principal of these were noticed by us in a recent number (Vol. XXXVIII, p. 396), we must refer to our former article for an account of the improved mode of practice recommended by him.

Dr. Mütter agrees with Mr. Liston in strongly reprobating the performance of "ovariotomy" in the present state of our knowledge of the diagnostic signs of ovarian dropsy. He here takes occasion also to introduce part of one of his own lectures, in which this opinion is expressed, with the candid and prudent reservation, "that should the difficulties about to be stated ever, by subsequent observation and research, be removed," he will be ready at once to change his present views, and rank himself among the advocates of the operation. Then follows a good summary of the most important objections that have been urged against the proposal.

Large, and we are inclined to think disproportionate, additions are made on the subjects of hemorrhage, cataract, anchylosis, club-foot, and division of tendons, which our limited space obliges us to refrain from noticing further.

Dr. Mütter's volume ends very abruptly. By way of completing the last subject (the treatment of calculous complaints), Mr. James Arnott is laid under contribution; and an account of the merits and principles of lithectasy, borrowed from that surgeon's paper, occupies rather more than ten pages. Unfortunately, Mr. Liston is not heard to the end; for his concluding lecture on lithotomy in the male, stone in the urethra, and on calculous and other diseases in the genito-urinary organs of the female is not here published. On referring to the 'Lancet,' we find that this lecture was reported in two parts, of which one appeared November 15th, the other December 13th, 1845; but neither, we suppose, reached Philadelphia in time for insertion in this volume. In his preface, dated January, 1846, Dr. Mütter says, "It will be observed that this volume contains all the lectures published up to the present date, but does not conclude the course. Another volume will be issued hereafter, should the publication of the lectures be continued." We should not have remarked on this little fact, had it not been a part only of the evidence the work affords of being composed with ill-judged haste; a haste which clearly originated in an anxiety on the part of the publisher to secure the profits of a promising speculation,—making it, as it would seem, quite a secondary consideration whether or not the work should be as worthy as possible of the two distinguished names under whose authority it appears. For this we do not conceive, nor would we for one moment be understood to imply, that the learned Professor of Jefferson College is answerable; but we cannot conscientiously acquit him of all responsibility. In default of a better and juster code of international law for protecting the fair interests and vested rights of authors (years of thought and study vested in print and paper), and defending them from the privateering system of the trade, we should like to see men of Dr. Mütter's character and position in the profession and in society, take their stand on the highest ground, and decline to append their names to hurried productions such as the present undoubtedly is. Nor is it expecting too much from them to do so, for lookers-on can see evident proofs of the ill effect the present system has upon their writings and upon themselves.

---

